# Possible vertebral brucellosis infection in a Neanderthal

**DOI:** 10.1038/s41598-021-99289-7

**Published:** 2021-10-06

**Authors:** Bruce Rothschild, Martin Haeusler

**Affiliations:** 1 Department of Vertebrate Paleontology, Carnegie Museum, 4400 Forbes Ave, Pittsburgh, PA 15213 USA; 2 Indiana University Health, Muncie, IN 47303 USA; 3 Institute of Evolutionary Medicine, University of Zürich, Winterthurerstr. 190, 8057 Zurich, Switzerland

**Keywords:** Evolution, Anthropology, Biological anthropology

## Abstract

The La Chapelle-aux-Saints 1 skeleton of an old (>60-year-old) male Neanderthal is renowned for the advanced osteoarthritis of its spinal column and hip joint, and their implications for posture and lifestyle in these Mid- to Late Pleistocene humans. Reassessment of the pathologic lesions reveals erosions at multiple non-contiguous vertebrae and reactive bone formation extending far beyond the left hip joint, which suggests the additional diagnosis of brucellosis. This implies the earliest secure evidence of this zoonotic disease in hominin evolution. Brucellosis might have been transmitted via butchering or eating raw meat and is well compatible with the range of prey animals documented for Neanderthals. The associated infertility could have represented an important aspect of health in these late archaic humans.

## Introduction

Review of the pathologic findings initially reported in the La Chapelle-aux-Saints 1 Neanderthal^1–5^ revealed alterations that appear attributable to the brucellosis. Today, brucellosis is globally the most prevalent zoonosis^6^. It was first described by David Bruce on the island of Malta in 1885^7^ and originally referred to as Malta fever. Evans^8^ provided the appellation brucellosis and its discovery in Malta is reflected in one of its species names, *Brucella melitensis*. Hughes^9^ suggests that Hippocrates described it in 1450 BCE. The first clinical description is attributed to Marston^10^. Other names derived from its symptoms [undulant fever and Lazybones disease (in China)], the veterinarian who identified its cause (Bang’s disease), the employment of those affected by it (Corps disease), its epidemiologic geography (undulant Mediterranean fever or gastric remittent fever, Neapolitan fever, Gibraltar or Rock fever, Cyprus fever, Maltese fever, Crimean fever)^11–14^.

Brucellosis is caused by an acid-fast non-motile, gram negative facultative intracellular coccobacillus related to animal (*Anaplasma, Bartonella, Rickettsia* and *Wolbachia*) and plant pathogens (*Agrobacterium, Ochrobactrum* and *Sinorhizobium*)^15,16^. Although it occurs worldwide, it is predominantly recognized in the Mediterranean region, Asia and Latin America^17^. There are at least 500,000 and perhaps 5–12 million new human cases each year^18,19^.

Brucellosis has a long history. The earliest occurrence was claimed in a 2.1–2.5-million-year-old *Australopithecus africanus* skeleton, StW 431^20^. A re-examination of the affected lumbar vertebrae L4 and L5 of StW 431 suggested, however, that the vertebral marginal lesions are more likely attributable to a limbus vertebra, i.e., an anterior disc herniation^21^. Other occurrences of brucellosis have been described in 3200–3000 BCE Bab edh-Dhra in Jordan^22^, 350 BCE–1200 CE Nubia^23^, second to fourth century BCE Butrint Albania^24^, and first century Herculaneum^25^. A study of mitochondrial genomes in early Bronze Age humans from the Novosvobodnaya North Caucasus site revealed *Brucella abortus*^26^. We now examine evidence for its presence in a Neanderthal.

## Results

**Figure 1:**
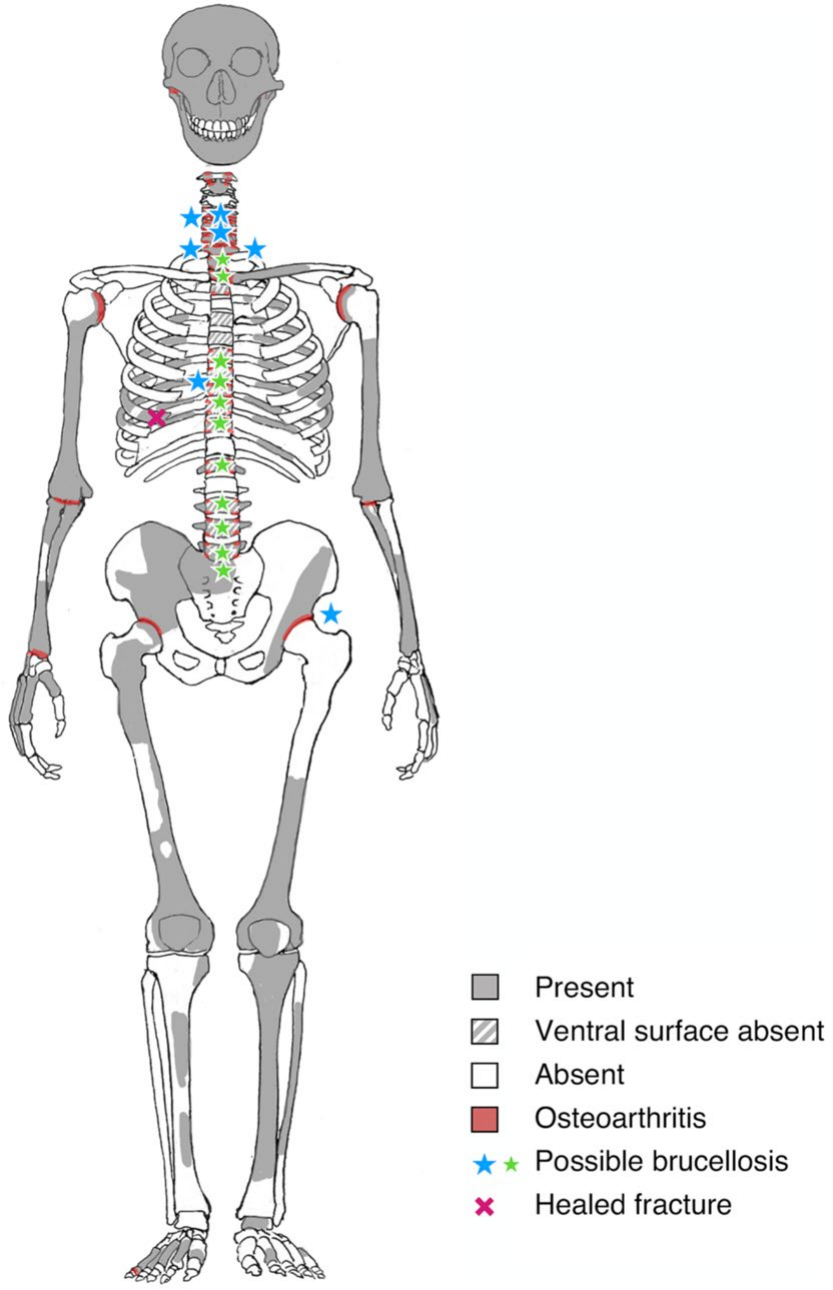
Preservation of the La Chapelle-aux-Saints 1 Neanderthal skeleton and distribution of the pathological lesions. Possible brucellosis leading to erosions affected the inferior endplates of vertebrae C5 and C6, the right facet joints C4/5 and C7/T1, the left transverse process T1, both facet joints T9/10, and the left hip bone (blue stars). Irregular grooves likely associated with granulomateous processes are found on the laminae of thoracolumbar vertebrae (green stars). Osteoarthritic changes (red) are present in the right temporomandibular joint, the facet joints of the vertebral column, glenohumeral joints, left sternoclavicular joint, elbow joints, right radiocarpal joint, hip joints and right fifth pedal proximal interphalangeal joint. A midthoracic right rib shows a healed fracture (cross).

All preserved vertebrae of La Chapelle-aux-Saints 1 showed mild to advanced osteoarthritic changes of the facet joints. Except for the cervical vertebrae, the ventral margin was damaged in all vertebral bodies. In the upper thoracic and the lumbar vertebrae, the ventral portions of the vertebral bodies were reconstructed by Boule^1^ with plasticine (Fig. 1). Nevertheless, the first two thoracic vertebrae were relatively well preserved. Similar to the lower cervical vertebrae, they only sustained minor damage (see also Refs.^3–5^). The ventral margin of the vertebral bodies was also reconstructed in all lumbar vertebrae. The preserved portions of vertebra L1 suggests however a pathological anteriorly wedge-shaped angle of 12° between the superior and inferior vertebral surface.

**Figure 2:**
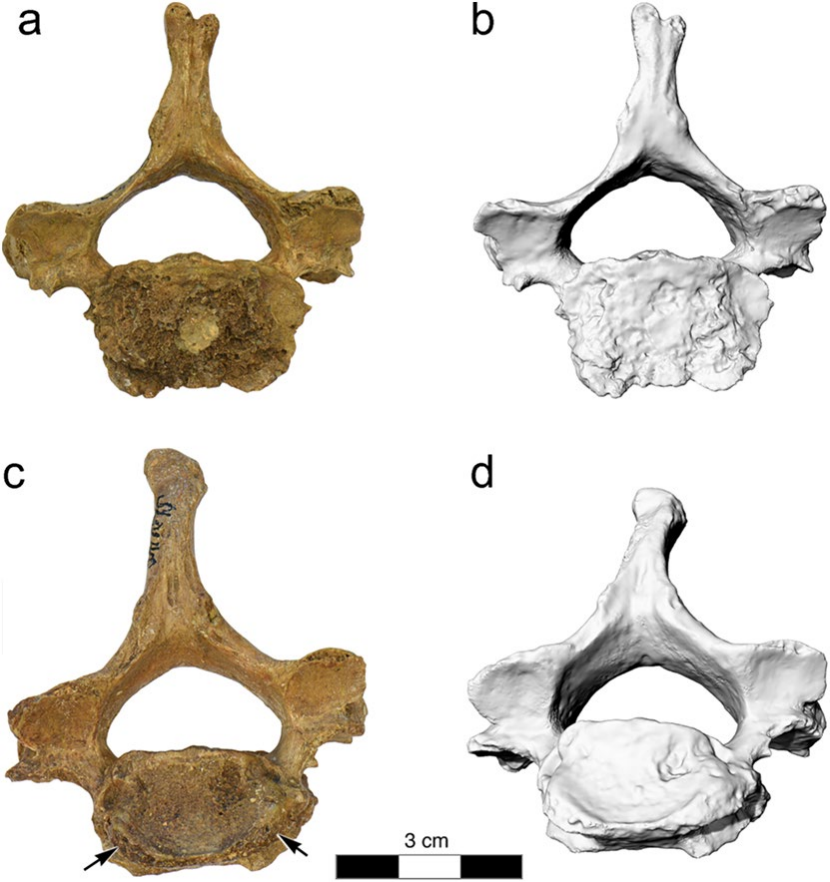
La Chapelle-aux-Saints 1 cervical vertebrae C5 and C6. (**a**,**b**) Photograph and 3D surface scan of vertebra C5, inferior view, (**c**,**d**) photograph and 3D surface scan of vertebra C6, inferior view. Note the marginal erosions (arrows).

Confluent vertebral endplate erosions with new bone formation were observed at the inferior vertebral surface of C5, which cannot be explained by osteoarthritis (Fig. 2). Anterior endplate grooves with smooth bases suggestive of marginal endplate erosions were also observed at the inferior vertebral surface of C6.

**Figure 3:**
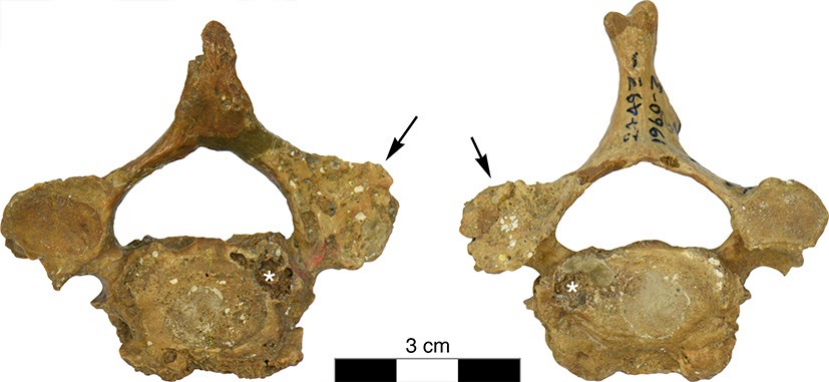
La Chapelle-aux-Saints 1 cervical vertebra 4, inferior view (left) and cervical vertebra 5, superior view (right). The right facet joint of C4/C5 is severely remodelled and shows multiple fine erosions (arrows). White asterisks (*) denote taphonomic damage to the vertebral surface.

**Figure 4:**
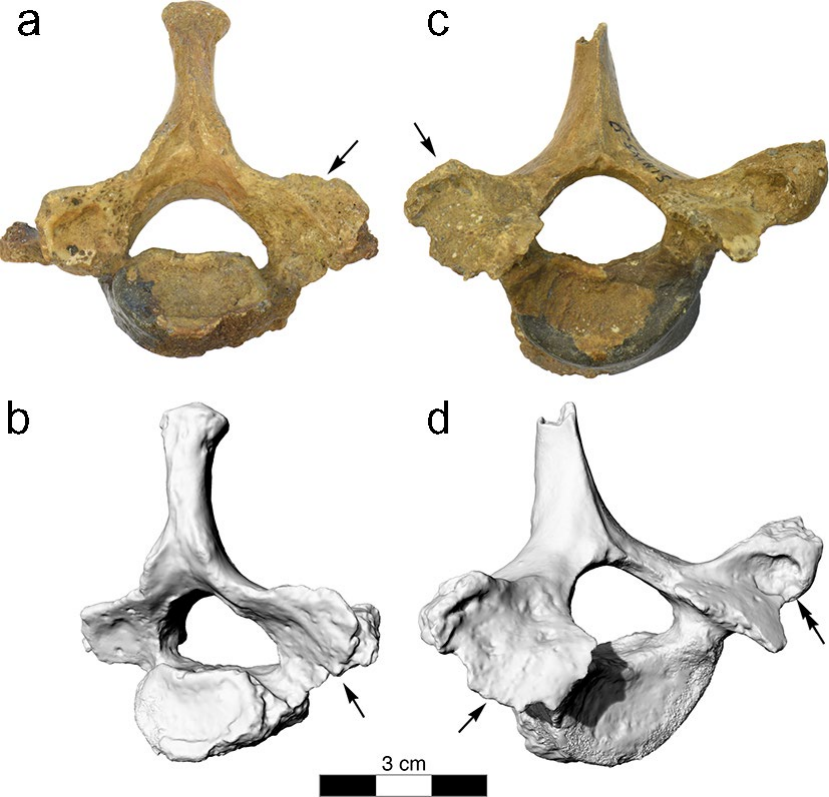
La Chapelle-aux-Saints 1 cervical vertebra C7 and thoracic vertebra T1. (**a**,**b**) Photograph and 3D surface scan of C7, inferior view, (**c**,**d**) photograph and 3D surface scan of T1, superior view. The right facet joint of C7/T1 is severely remodelled and shows multiple fine erosions (arrows). The transverse process of T1 shows a large erosive defect suggestive of a space-occupying granulomatous process (double arrows).

**Figure 5:**
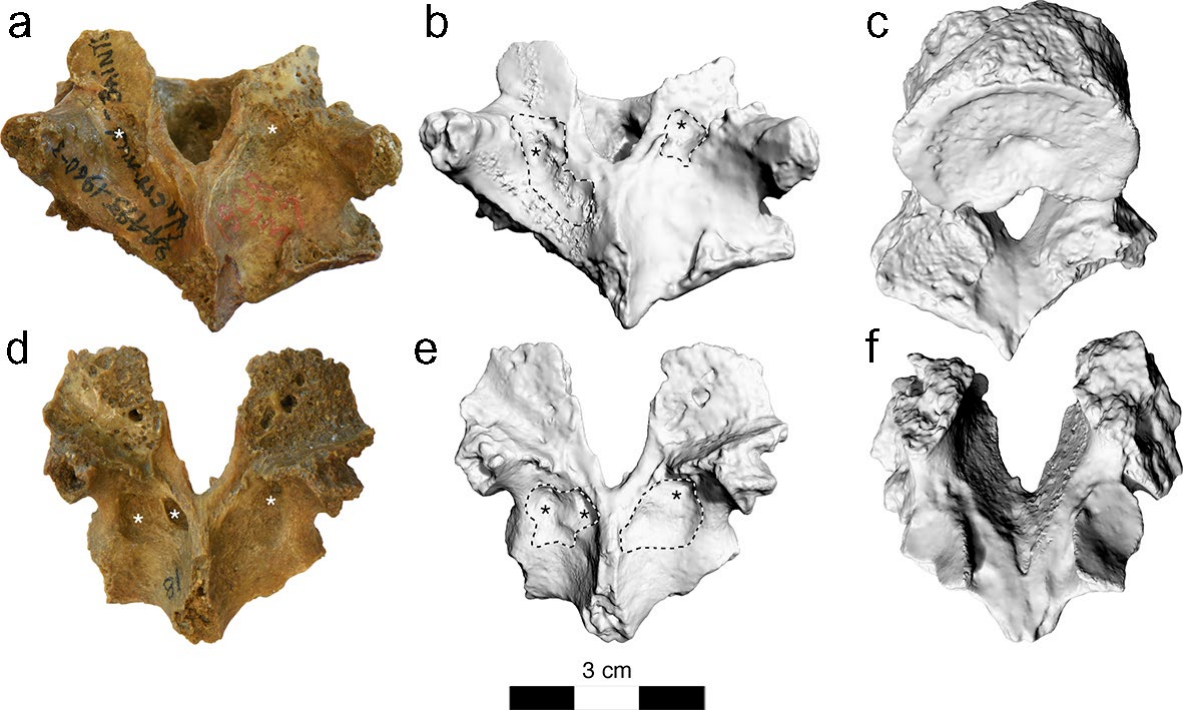
La Chapelle-aux-Saints 1 thoracic vertebrae 9 and 10, showing erosion of the facet joints T9/T10. (**a**) photograph of T9, dorsal view; the left superior facet joint of T9 is unaffected, while the right superior facet joint shows osteoarthritic changes with eburnation; the left inferior facet joint of T9 is broken off, the right inferior facet joint is severely remodelled and shows multiple fine erosions, (**b**) 3D surface scan of T9, dorsal view, (**c**) 3D surface scan of T9, oblique inferior view. (**d**) photograph of T10, dorsal view, (**e**) 3D surface scan of T10, dorsal view, (**f**) 3D surface scan of T10, oblique inferior view; the inferior facet joints of T10 are unaffected. Possible erosive defects caused by space-occupying granulomatous processes are marked with asterisks and dashed lines along their contours on the 3D scans.

**Figure 6:**
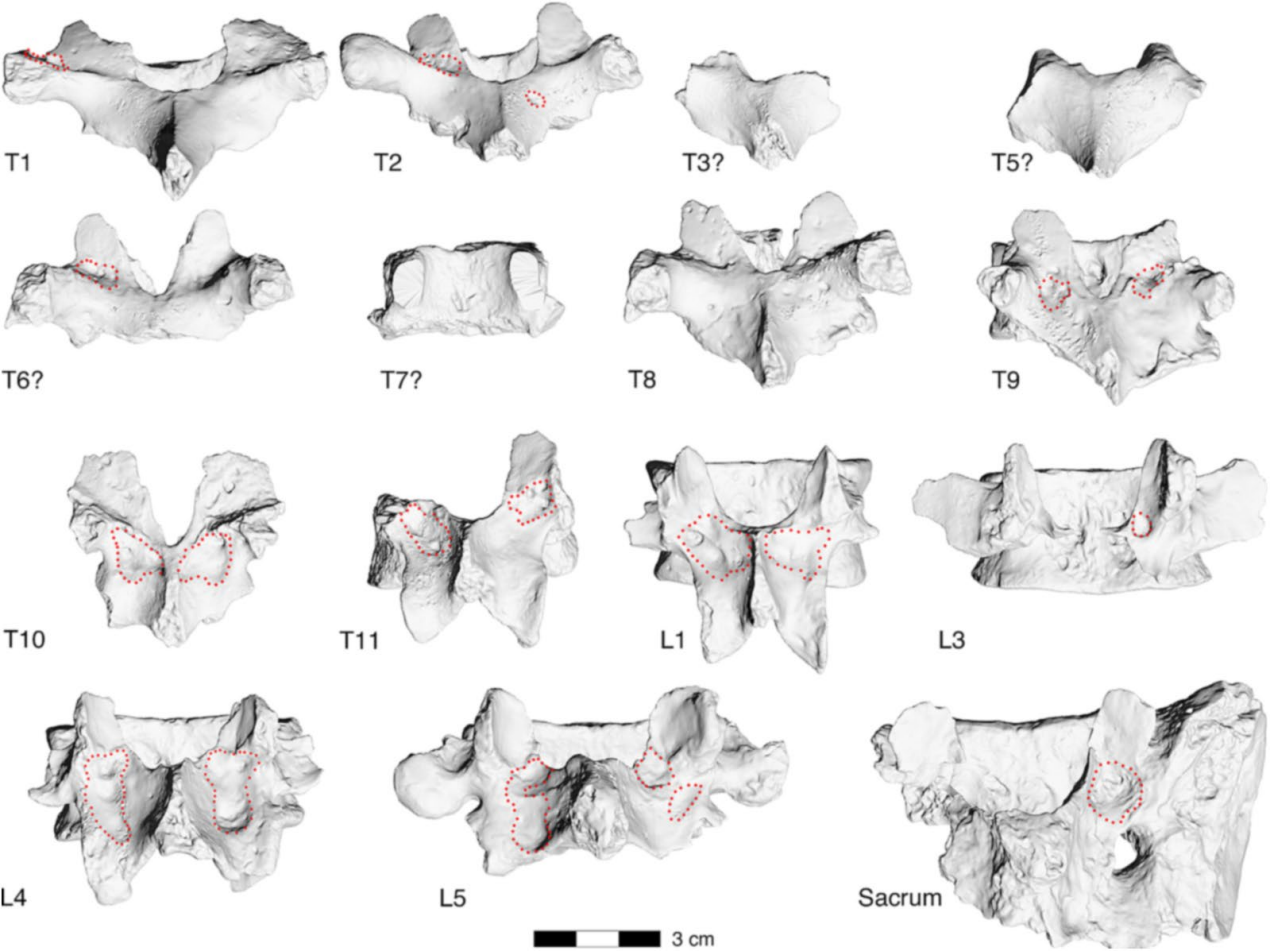
3D surface scans of vertebrae T1 to L5 and the sacrum, dorsal view. Possible erosive defects caused by space-occupying granulomatous processes are marked with dotted lines along their contours on the 3D scans. Note that we have virtually disassembled the lamina possibly belonging to vertebra T5 and the vertebral body possibly belonging to T7 that have erroneously been joined by Boule^1^ with plasticine.

Similar erosions were observed at the right facet joint C4/C5 (Fig. 3), the right facet joint C7/T1 (Fig. 4), and T9/T10 (Fig. 5). Moreover, the left transverse process of T1 showed an irregular 11.2 × 8.1 mm large and 3.2 mm deep lytic lesion with smooth margins (Fig. 4). Irregular grooves with intact cortical bone and smooth margins were also found immediately adjacent to the left superior articular facet and in the middle of the right lamina of vertebra T2, as well as in the laminae of all preserved thoracolumbar vertebrae between T9 and L5 as well in S1 (Fig. 6). The irregular morphology of these grooves suggest that they are caused by nodular, multi-lobulated granulomateous processes. The depressions measure up to 20 × 9 × 3.5 mm (e.g., in L4) and are thus more extended, deeper and more jagged than imbrication pockets that are associated with hyperextension of the spine and also occur immediately adjacent to the superior articular facets^27^.

**Figure 7:**
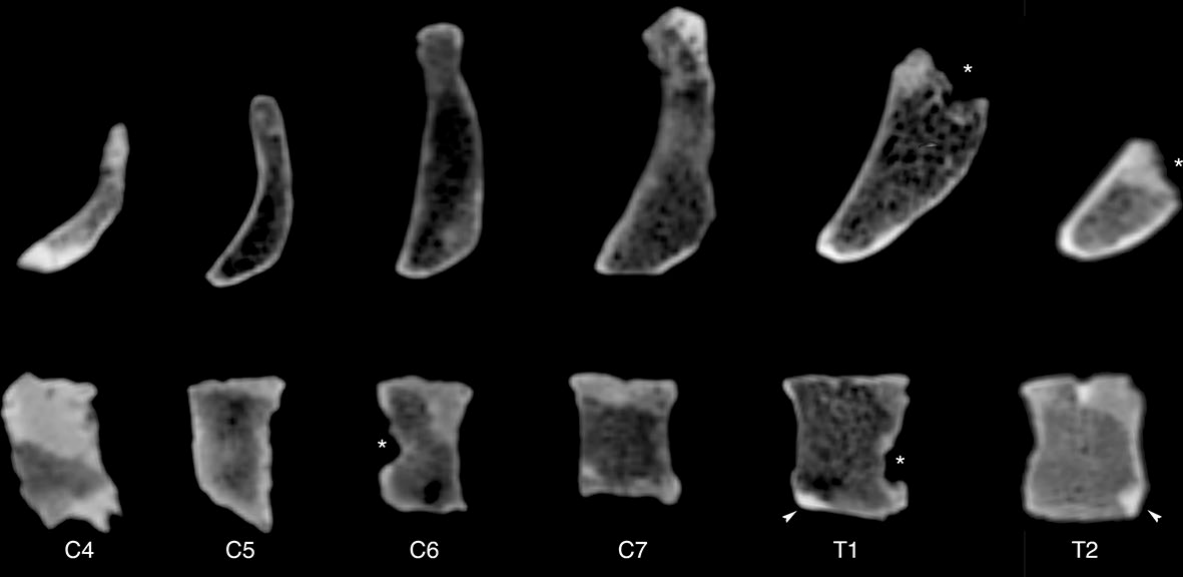
Midsagittal CT sections of vertebrae C4 to T1. Note the patchy mineralisation of the fossil vertebrae. Probable taphonomic defects are marked by asterisks. The anterosuperior and the anteroinferior rims of vertebral bodies T1 and T2, respectively, have been reconstructed in plasticine (arrowheads).

Another type of depressions represent Schmorl’s nodes that can be recognized in the centres of the inferior vertebral body surface of T6 and on both the superior and inferior surfaces of T7–T9 (see Ref.^21^). Given the relatively low resolution of the medical CT scans of the La Chapelle-aux-Saints 1 skeleton compared to µCT scans, they were not informative save for localizing the pathological lesions (Fig. 7).

**Figure 8:**
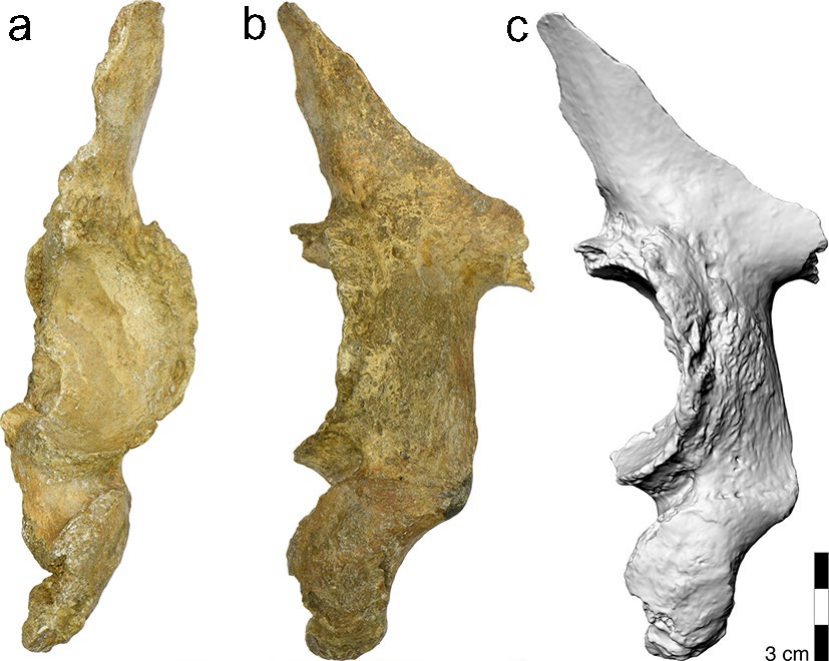
The partial left hipbone of La Chapelle-aux-Saints 1. (**a**) photograph in lateral view, (**b**) posterior view. (**c**) 3D surface scan, oblique posterior view. Note the presence of erosions and new bone formation that extends far beyond the hip joint. This does not occur in osteoarthritis, but is compatible with extra-articular granulomas in brucellosis.

The left hip bone is fragmentary (Fig. 8). The articular surface in the weight-bearing posterosuperior portion of the acetabulum is eburnated. The acetabular margin bears large osteophytes and shows reactive new bone formation with erosions extending to the extraarticular portion of the lower ilium and upper ischium. The hip joint is, however, not deformed and shows no indication of hip dysplasia or hip joint luxation. The left proximal femur is not preserved. The right hip joint showed moderate osteoarthritic changes. Eburnation of the subchondral bone was also observed in both proximal humeri.

## Discussion

The possibility of osteoarthritis has been considered as an explanation for the vertebral and hip joint lesions observed in the La Chapelle-aux-Saints 1 Neanderthal^2–4^. More precisely, La Chapelle-aux-Saints 1 was thought to have suffered from both osteoarthritis and spondylosis deformans, as the term osteoarthritis is reserved for the disease of diarthrodial joints, while that appellation has been replaced by the term spondylosis deformans for the intervertebral disk articulations^28–30^. Both spondylosis deformans and osteoarthritis are recognized by production of osteophytes and subchondral sclerosis^31–35^. In addition, advanced osteoarthritis is characterised by subchondral cyst formation and smoothing or eburnation of joint surfaces^36^. However, eburnation simply identifies sufficient cartilage loss to allow bone to rub on bone. Any pathology that results in cartilage loss, be it mechanical (e.g., osteoarthritis) or inflammatory (e.g., infection or spondyloarthropathy) can produce eburnation^28^.

Spondylosis and facet joint osteoarthritis as well as osteoarthritis of the peripheral joints affecting both glenohumeral joints, both hip joints and possibly also the elbow and radiocarpal joints can undoubtedly be observed in La Chapelle-aux-Saints 1, as described earlier^1–5,21^.

Yet, not all damage can be attributed to osteoarthritis. Features that are incompatible with the diagnosis of osteoarthritis and spondylosis deformans include the presence of actual subchondral and marginal erosions at the inferior endplates of vertebrae C5 and C6, erosions in the right facet joints C4/5 and C7/T1 as well as both facet joints T9/10, lytic lesions in the extra-articular areas of T1 to S1, and erosions and reactive bone formation extending far beyond the left hip joint^28,30^. Rather, the mixed picture of destructive and proliferative processes is typical of an inflammatory aetiology. Although the term erosion has also been applied to the cartilage damage that osteoarthritis produces, this damage is characterized by mechanical abrasion and fissures and not by the inflammatory process that produce erosions in rheumatologic disorders or chronic infection^28,30,35,37^. Rheumatologic disorders that mainly affect the spine are classified as spondylarthropathies. They include ankylosing spondylitis, reactive arthritis (previously referred to as Reiter’s syndrome), psoriatic arthritis and enteropathic arthritis associated with inflammatory bowel diseases. The scattered distribution of the erosive lesions along the axial skeleton of La Chapelle-aux-Saints 1 and the presence of extensive granulomateous processes suggest, however, a chronic infectious or tumorous aetiology. Osteolytic tumours typically grow from the vertebral body into the appendicular regions and preferentially destroy the anterior and posterior edges of the vertebral bodies rather than the endplates^38^. Chronic infections producing spondylitis and spondylodiscitis, on the other hand, mostly include tuberculosis and brucellosis. Other infections like actinomycosis, blastomycosis, coccidiodomycosis, and echinococcosis are extremely rare and mainly lead to spondylitis and spondylodiscitis in immunodeficient patients^38^. Tuberculosis, on the other hand, typically causes a wedge-shaped destruction of one or two adjacent vertebral bodies leading to the pathognomonic gibbus deformity, which is most often located in the mid-thoracic region. In the La Chapelle-aux-Saints 1 skeleton, vertebra L1 is slightly anteriorly wedge-shaped with an angle of 12° between the superior and inferior vertebral surface, but there are no indications of destructive processes in that vertebra. Rather, the wedge-shaped vertebral body of L1 might have resulted from a healed vertebral fracture or from Scheuermann’s disease (for diagnostic criteria see Refs.^39,40^).

The pattern of the erosive defects observed in the La Chapelle-aux-Saints 1 vertebrae and the reactive new bone formation at the left hipbone is, however, characteristic of brucellosis infection. Thus, vertebrae are involved in 2–53% of brucellosis infections^17,41,42^. Typically, the disease initially affects a single vertebral endplate, mostly in the lumbar spine, but it can also be observed at multiple levels, affecting non-contiguous endplates and facet joints also in the thoracic and cervical spine^17,42^. In addition, monoarthritis or oligoarthritis may occur, with the knee and hip joints being most commonly affected^12^. The lesions have a specific erosive character and a location that is seemingly pathognomonic for the disease, initially manifesting as an osteolysis/groove on the anterior aspect of superior vertebral endplates^17,43–46^. Specifically referred to as Pedro i Pons’ sign^47,48^, such grooves are not found with pyogenic (e.g., *Staphylococcus*) vertebral infections^49^ or tuberculosis^28^. Unfortunately, this characteristic destruction of the anterosuperior margin of the lumbar vertebrae cannot be observed in La Chapelle-aux-Saints 1 due to taphonomic damage of the anterior portion of all thoraco-lumbar vertebrae.

However, the well-preserved vertebrae C4 to T2 of La Chapelle-aux-Saints 1 show several pathological lesions suggestive of brucellosis. Examination of vertebrae C5 and C6 revealed erosive changes to the inferior vertebral surface (Fig. 2) that do not occur in spondyloarthropathy^28,30,50^. The smooth character of the base of the groove in C6 evidences reactive new bone formation and not simply taphonomic bone loss (which would have exposed trabecular bone). Confluent erosion with new bone formation is quite different from the edge (Romanus) lesions noted with spondyloarthropathy^28,51^. That appellation refers to apophyseal ring erosion, producing loss of the edge of the endplate in spondyloarthropathy, in contrast to production of a groove on its subchondral (disk-facing) surface. Such vertebral endplate grooves, however, are quite characteristic of the damage caused by brucellosis^17,43–48^. In later stages of the infection, the erosive changes gradually expand over the entire vertebral endplate, producing the condition seen in vertebra C5.

Notation of erosions of the right facet joint of C4/C5 and C7/T1 and both facet joints of T9/T10 (Figs. 3, 4, 5) suggested differential diagnostic consideration of spondyloarthropathy or granulomatous infection. Facet joint involvement is found in up to one-third of individuals with brucellosis^6,44,52,53^. However, such erosions of the facet joints and vertebral endplate are not anticipated with spondyloarthropathy^28,30,50^. In addition, the transverse process of T1 and the laminae of the lower thoraco-lumbar vertebrae show large erosions characteristic of space-occupying granulomatous processes (see Figs. 4, 5 and 6)^28,54–56^. The latter are caused by infectious processes that may be attributed to tuberculosis, fungal disease and brucellosis^28,30^. Fungal disease produces erosions that do not respect variation in bone density, preserving a spheroid shape^54^. Tuberculosis is characterized by zones of bone resorption^30,56^. Neither pattern is observed in the La Chapelle-aux-Saints 1 Neanderthal.

The severe arthritic changes at the left hip joint have previously been attributed to trauma^3^, but there is otherwise no evidence for trauma. The presence of erosions and new bone formation suggest that on top of the age-related primary osteoarthritis that might initially have been present (and which can also be observed in the right hip joint and both humeri), a secondary phenomenon, i.e., hip joint infection by brucellosis occurred. In fact, osteoarthritis-related changes would be confined to the joint region itself and do not extend to the extra-articular region as it is the case of La Chapelle-aux-Saints 1. Inflammatory processes that extend beyond the joint region are, however, not unusual in brucellosis, analogous to the paraspinal granulomas probably present in vertebrae T1 and T10 (see also Ref.^12^).

Neanderthals likely were infected by brucellosis during butchering of prey animals, not unlike the abattoir experience today^13^, or by eating raw meat. The main causative organisms of brucellosis have been found in a wide range of wildlife. Particularly, *Brucella melitensis* has been reported in ibex and chamois*,* while *B. abortus* and *B. suis* have a preference for most other bovines and suids, including wild sheep, goats, wild cattle, European bison, reindeer, roe and red deer, wild boar, but also horses, hares and marmots^57–61^. All these animals were important components of the Neanderthal diet^62–64^. Only the two largest Neanderthal prey animals, mammoths and woolly rhinoceros, might not have been reservoirs for *Brucella* as inferred from their extant relatives^65–67^. The host preference of the different *Brucella* species might suggest that La Chapelle-aux-Saints 1 was infected by *B. abortus* rather than *B. melitensis*. While the latter species often leads to acute pain and immobilization, *B. abortus* is known to cause a milder course of the disease. This might therefore explain the advanced stage of the arthritis in La Chapelle-aux-Saints 1 and his survival to the advanced age of perhaps well over 60 years^5^.

Symptoms of brucellosis not only include fever, arthritis, endocarditis, neurologic defects, spinal deformity, reduced milk production, but also epididymitis/orchitis, infertility, still births and abortions^68^. This could have represented an important aspect of health in Neanderthals, or more generally in Palaeolithic humans.

## Materials and methods

The La Chapelle-aux-Saints 1 burial of an old male Neanderthal was discovered in 1908 in a pit in the Bouffia Bonneval cave near the village of La Chapelle-aux-Saints in south-western France^1^, together with dental remains of four additional individuals. Electron spin resonance (ESR) dating suggested a geological age of 47 ka ± 3 ka BP (early U-uptake model) or 56 ka ± 4 ka BP (linear U-uptake model)^69^. The auricular surface of the ilium suggests an individual age of 62 ± 13 years^5^. The partial skeleton preserves an almost complete skull with extensive post-canine tooth loss, a scapular fragment, portions of all long bones and both hipbones, as well as 19 variously complete vertebrae and a partial sacrum. The vertebrae include C1 and C2, C4 to T2, two fragmentary upper thoracic vertebrae (possibly T3 or T4 and T6 or T7), T8 to T12, L1, and L3 to S2^5,70^ (Fig. 1).

The vertebrae of the La Chapelle-aux-Saints 1 Neanderthal were examined macroscopically and at 10 × electronic magnification to characterize the presence and nature of vertebral endplate and facet alterations. In addition, CT scans of the vertebrae performed by the Musée de l’Homme were examined. All fossil bones were also surface scanned using a high-resolution surface scanner (PT-M4c, Polymetric GmbH, Darmstadt, Germany)^71^.

The right hipbone is currently missing, and a cast as well as old photographs (curtesy of Erik Trinkaus) were examined.

## Data Availability

All data analysed during this study are included in this published article. The La Chapelle-aux-Saints 1 skeleton is housed at the Musée de l’Homme, Paris.
